# Collective Motional Temperature

**DOI:** 10.1021/acsomega.6c01599

**Published:** 2026-05-08

**Authors:** Sohila Abdelhafiz, Amir M. Jazayeri, Aristide Dogariu

**Affiliations:** † CREOL, The College of Optics and Photonics, 6243 University of Central Florida, 4304 Scorpius Street, Orlando, Florida 32816, United States; ‡ The Department of Physics, University of Central Florida, 4111 Libra Drive, Orlando, Florida 32816, United States

## Abstract

We introduce the concept of collective motional temperature and analyze its time evolution in a dense system of interacting nanoparticles. Using an experimental approach that provides the spatial and temporal resolution necessary to study nonequilibrium phenomena in large-scale dynamic systems, we examine the colloidal medium under the simultaneous influence of gravity and an external optical field. The optical field is dynamically modified by the colloidal particles and therefore mediates their many-body interactions. We find that the time evolution of the number density of the particles is mainly affected by the action of gravity and radiation pressure, while the collective motional temperature is determined by the dynamic optical speckle field. We show that, in agreement with recent studies on one isolated particle, the effective heating and cooling times are not equal for an interacting many-body system, either. We also show how the collective motional temperature depends on the intensity of the external optical field.

## Graphical abstract

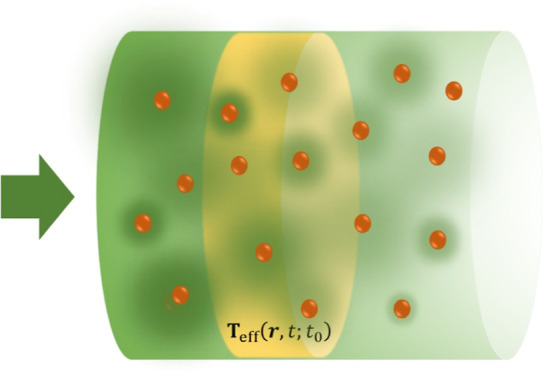

## Introduction

An open system in contact with some reservoirs and under the influence of nontime-varying external drives usually reaches a steady state. This is the situation where the density matrix (i.e., the matrix consisting of the transition probabilities between the states of the otherwise closed system) does not change in time anymore. For a physical system consisting of massive particles, e.g., electrons or dielectric particles, to which position is attributable, a manifestation of a steady state is that the local density of the particles does not change in time. The transition from an initial to a final steady state of the system is a nonequilibrium and nonsteady-state process. Recently, there has been an immense interest in the dynamics of the heating and cooling processes in both classical and quantum systems.^1–7^ Unlike many of such studies, we examine here a many-body system^8^ for which collective effects have a decisive role.

The physical system under scrutiny is a dense colloid of dielectric particles, which is under the influence of both gravity and a coherent laser beam that illuminates the medium laterally. A schematic is depicted in Figure 1. For each particle, the time evolution of its position can be described by an overdamped Langevin equation:

1 $$M\Gamma {\dot{\vec{r}}}_{m}=\mathrm{x̂}\left(M-M_{w}\right)g+{\mathrm{f⃗}}_{rp,m}+{\mathrm{f⃗}}_{th,m}+{\mathrm{f⃗}}_{sp,m}$$

where *x̂* denotes the unit vector parallel to the *x* axis, the subscript “m” is the particle’s index, and M and Γ denote its mass and damping rate, respectively. The mass of displaced water is such that M_w_/M <1. We shall explain each term in eq 1.

**Figure 1:**
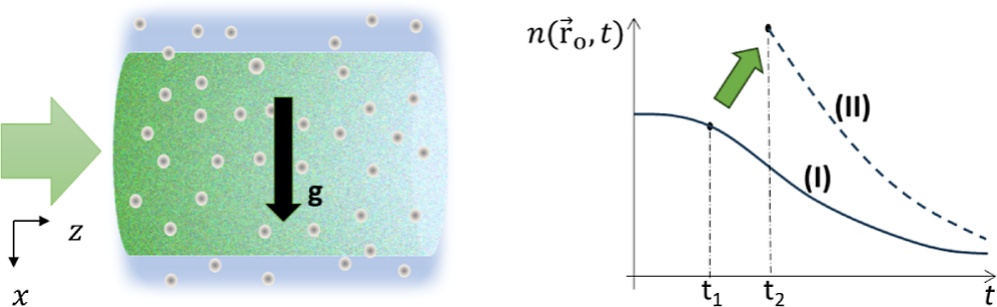
Particulate system under simultaneous influence of gravity and an optical field. Due to the scattering of light by the particles, the optical field (green) consists of a coherent part decaying along *z* and a time-varying speckle component. The optical field affects the particles’ dynamics, which, in turn, influences the field’s properties. The effects of the optical field and gravity are intertwined in a nonlinear way leading to a complex evolution of the local particle number density. At a given observation point ${\mathrm{r⃗}}_{o}$ , $n\left({\mathrm{r⃗}}_{o},t\right)$ evolves differently in case I and case II: the external laser beam is not turned on in case I, while it is turned on (at t_1_) and off (at t_2_) in case II. The different behavior is maintained even for large *t* and even if both cases have the same initial distribution n(r⃗,0) .

Stokes’ law yields a good approximation for the damping rate Γ = 6πRη/M in terms of the particle’s radius R and mass M as well as water’s dynamic viscosity η. From a microscopic viewpoint, the damping force $-M\Gamma {\dot{\vec{r}}}_{m}$ and the thermal random force ${\mathrm{f⃗}}_{th,m}$ are both due to collisions between the particle and water molecules. As ${\mathrm{f⃗}}_{th,m}$ varies fast in comparison with the time resolution of usual measurements, the correlation ⟨*f*_th,m,i_(*t*)*f*_th,m,i_(*t*′)⟩ is typically assumed to be a Dirac delta function K_th_δ­(*t*–*t*′), where the index “i” defines the force components (“*i*” is *x*, *y*, or *z*), and K_th_ is a constant. Also, the correlations ⟨*f*_th,m,i_(*t*)*f*_th,m,j_(*t*′)⟩ are assumed to vanish for *i*≠*j*. As in the absence of gravity and the laser beam, each component of the particle’s position ${\mathrm{r⃗}}_{m}$ must obey the fluctuation–dissipation theorem, the constant K_th_ must be equal to 2MΓ*k*_B_T_0_ in terms of the damping rate Γ and water’s temperature T_0_, where k_B_ denotes the Boltzmann constant.^9,10^ In the following, we will present a simplified description of the complex physical problem illustrated in Figure 1.

With respect to the observation time, the optical force acting on particle “*m*” can be decomposed into slow ${\mathrm{f⃗}}_{rp,m}$ and fast ${\mathrm{f⃗}}_{sp,m}$ components. The term ${\mathrm{f⃗}}_{rp,m}$ refers to the radiation pressure exerted by the part of the optical fields that maintains the phase profile of the incident laser beam. Sometimes this field component is called “coherent” field. When the size of the incident beam is much larger than the wavelength, one can consider that ${\mathrm{f⃗}}_{rp,m}={ẑf}_{rp,m}$ . Due to the scattering by the particles, the optical power *P*_coh_ carried by the coherent field decays upon propagation according to the Beer–Lambert law, $P_{coh}=P_{0}e^{-2z/l_{s}}$ .^11,12^ One might conclude that *f*_rp,m_ acting on the particle “*m*” is $f_{0}e^{-2z_{m}/l_{s}}$ , where f_0_ denotes the optical force exerted on that particle in the absence of all other particles. However, the implicit assumption in the Beer–Lambert law is that the particles are distributed uniformly. Therefore, in a more precise description, the force $f_{rp}\left(\mathrm{r⃗},t\right)$ should be regarded as a functional $\psi \{\mathrm{r⃗};n({\mathrm{r⃗}}^{\prime },t)\}$ , where $n\left({\mathrm{r⃗}}^{\prime },t\right)$ denotes the particle number density at ${\mathrm{r⃗}}^{\prime }$ and *t*.

The second component of the optical field acting on the ensemble of particles, ${\mathrm{f⃗}}_{sp,m}$ , is a three-dimensionally random speckle field that has no phase relationship with the incident beam. Importantly, this speckle field is not constant in time because it is generated by scattering from moving particles.^13^ We note that the optical force exerted by a static speckle field on a particle depends on the particle’s position but it has no explicit time dependence, whereas ${\mathrm{f⃗}}_{sp,m}$ due to our time-varying speckle field has an explicit time dependence. In this sense, the situation is akin to that of a particle placed in a time-dependent random potential.^14–17^

To estimate how fast ${\mathrm{f⃗}}_{sp,m}$ changes in time, we note that a considerable change in ${\mathrm{f⃗}}_{sp,m}$ requires a notable change in the speckle field and therefore a considerable reconfiguration of the particles. An estimation of the time that it takes for a particle to have an overdamped motion equal to its radius in a random direction in the absence of gravity and external field is on the order of R^2^/D_E_, where the diffusion coefficient D_E_ is equal to D_E_ = k_B_T_0_/(MΓ).^18^ For our experiments, this time is around 5 ms. The correlation $\left\langle f_{sp,m,i}\left(\mathrm{r⃗},t\right)f_{sp,m,i}\left(\mathrm{r⃗},t^{\prime }\right)\right\rangle$ of each component of the exerted ${\mathrm{f⃗}}_{sp,m}$ is considered to be $\left[K_{sp,m}\left(\mathrm{r⃗},t\right)/\tau_{sp}\left(t\right)\right]e^{-2\left|t-t^{\prime }\right|/\tau_{sp}\left(t\right)}$ , where τ_sp_ denotes the reconfiguration time of the speckle field, which is estimated to be around 0.5 ms in the conditions of our experiment. As the speckle dynamics fluctuates much faster than the particle’s motion, the correlation $\left\langle f_{sp,m,i}\left(\mathrm{r⃗},t\right)f_{sp,m,i}\left(\mathrm{r⃗},t^{\prime }\right)\right\rangle$ can be approximated to be a Dirac delta function $K_{sp,m}\left(\mathrm{r⃗},t\right)\delta_{ij}\delta \left(t-t^{\prime }\right)$ . We will return to this point later.

## Results and Discussion

The local number density n(r⃗,t) of particles is in practice defined with respect to a region centered at *r⃗* and of a volume V_o_ much smaller than the total volume and, of course, larger than the volume of a particle. Theoretically speaking, one may approximate n(r⃗,t) by $\sum_{m}p_{m}\left(\mathrm{r⃗},t\right)$ , where $p_{m}\left(\mathrm{r⃗},t\right)\;dV$ is the probability of finding the center of the particle “m” at time *t* within an infinitesimal volume d*V* centered at *r⃗*. As the particles interact with each other via their scattered optical fields, the position of each particle as governed by eq 1 is, in fact, not a three-dimensional Markov process. However, one can consider a self-consistent approach, which in this sense is similar to mean field approximations in the study of quantum many-body systems, to provide a simple description of a complex problem. The influence of the state of other particles (i.e., their positions and velocities) on the particle “*m*” can be accounted for in $\left\langle f_{sp,m,i}\left(\mathrm{r⃗},t\right)f_{sp,m,i}\left(\mathrm{r⃗},t^{\prime }\right)\right\rangle$ . We can derive a master equation [i.e., an equation relating $\partial p_{m}\left(\mathrm{r⃗},t\right)/\partial t$ for one *r⃗* to $p_{m}\left({\mathrm{r⃗}}^{\prime },t\right)$ for all other ${\mathrm{r⃗}}^{\prime }$ ] from eq 1.^19^ The corresponding Fokker–Planck equation is found to be

2 $$\frac{\partial p\left(\mathrm{r⃗},t\right)}{\partial t}=-\sum_{i=1}^{3}\frac{\partial }{\partial x_{i}}\left[A_{i}\left(\mathrm{r⃗},t\right)p\left(\mathrm{r⃗},t\right)\right]+\sum_{i=1}^{3}\sum_{j=1}^{3}\frac{\partial^{2}}{\partial x_{i}\partial x_{j}}\left[D_{ij}\left(\mathrm{r⃗},t\right)p\left(\mathrm{r⃗},t\right)\right]$$

where *D*_*ij*_ = *D*δ_*ij*_, $D=k_{B}\left[T_{0}+\Delta T_{eff}\left(\mathrm{r⃗},t\right)\right]/\left(M\Gamma \right)$ , and

$$A_{1}=\left[\left(M-M_{w}\right)g+0.5\partial \Delta T_{eff}\left(\mathrm{r⃗},t\right)/\partial x_{1}\right]/\left(M\Gamma \right)$$

$$A_{2}=\left[0.5\partial \Delta T_{eff}\left(\mathrm{r⃗},t\right)/\partial x_{2}\right]/\left(M\Gamma \right)$$

$$A_{3}=\left[f_{rp}\left(\mathrm{r⃗},t\right)+0.5\partial \Delta T_{eff}\left(\mathrm{r⃗},t\right)/\partial x_{3}\right]/\left(M\Gamma \right)$$

Here, $\Delta T_{eff}\left(\mathrm{r⃗},t\right)=K_{sp}\left(\mathrm{r⃗},t\right)/\left(2M{\Gamma k}_{B}\right)$ can be regarded as a change in the motional temperature of the particles. Note that we have adopted the Stratonovich convention^20^ for the time integration of ${\mathrm{f⃗}}_{sp,m}$ . Also, note that we have dropped the subscript “*m*” that shows the indices of the particles because all $p_{m}\left(\mathrm{r⃗},t\right)$ are governed by eq 2, the only difference being in their initial conditions $p_{m}\left(\mathrm{r⃗},0\right)$ . In the absence of gravity and the laser beam, eq 2 reduces to the well-known diffusion equation, $\partial p\left(\mathrm{r⃗},t\right)/\partial t=D\nabla^{2}p\left(\mathrm{r⃗},t\right)$ , where the diffusion coefficient D is equal to D_E_ = k_B_ *T*_0_/(MΓ).^18^

We will now discuss two aspects of eq 2. For the sake of simplicity, let us assume that Δ*T*_eff_ is zero, and *f*_rp_ does not depend on the observation time and position. Even under these simplifying assumptions, the $p_{m}\left(\mathrm{r⃗},t\right)$ found in the presence of gravity and radiation pressure (i.e., g ≠ 0 & *f*_rp_ ≠ 0) is not a linear superposition of the no-gravity case (i.e., g = 0 & *f*rp ≠ 0) and the no-radiation-pressure case (i.e., g ≠ 0 & *f*_rp_ = 0). However, it should be noted that the expectation value of ${\mathrm{r⃗}}_{m}$ in the presence of gravity and radiation pressure is a linear superposition of the expectation values for the no-gravity case and the no-radiation-pressure case, assuming that the initial condition $p_{m}\left(\mathrm{r⃗},0\right)$ is δ(r⃗) in all the three cases.

The second subtlety regarding eq 2 is the initial condition itself. Let us assume that the particle “*m*” is initially located at a known position, i.e., $p_{m}\left(\mathrm{r⃗},0\right)=\delta \left(\mathrm{r⃗}-{\mathrm{r⃗}}_{init,m}\right)$ . For simplicity, we still assume that Δ*T*_eff_ is zero, and *f*_rp_ does not depend on the observation time and position. Let us consider two scenarios: (i) the laser beam is never turned on and (ii) the laser beam is turned on at *t* = t_1_ and then turned off at *t* = t_2_, while gravity is present in both scenarios. For any given observation point $\mathrm{r⃗}={\mathrm{r⃗}}_{o}$ , $p_{m}^{I}\left({\mathrm{r⃗}}_{o},t\right)$ and $p_{m}^{II}\left({\mathrm{r⃗}}_{o},t\right)$ are identical for 0 < *t* < t_1_, and start to differ for *t* > t_1_. However, it is almost impossible to find a time t_3_ > t_2_ such that the evolutions of *p*_m_^I^ and *p*_m_^II^ again become identical for *t* > t_3_. As a result, the evolutions of $n^{I}\left({\mathrm{r⃗}}_{o},t\right)\approx \underset{m}{\sum }p_{m}^{I}\left({\mathrm{r⃗}}_{o},t\right)$ and $n^{II}\left({\mathrm{r⃗}}_{o},t\right)\approx \sum_{m}p_{m}^{II}\left({\mathrm{r⃗}}_{o},t\right)$ will never be the same even after a time much larger than *t*_2_. This point is illustrated in the right panel of Figure 1.

Let us now focus our attention on Δ*T*_eff_. The notion of effective motional temperature has so far been the subject of many studies on nonequilibrium systems.^14,20,21^ One example is a particle that moves under the influence of two thermal baths with different temperatures.^22^ Another example is a particle with a motional (or vibrational) degree of freedom coupled to an optical field increasing or decreasing the motional (or vibrational) temperature.^23^ The third example is an active Brownian particle, where the chemical reactions happening on its surface cause it to experience random kicks and therefore higher effective temperature and diffusion coefficient.^24–27^ Notably, all these instances are single-particle effects and, therefore, fundamentally different from our physical situation.

The fourth and last example, which might seem most similar to our problem, is a Brownian particle that, under the influence of a two-dimensional static speckle field, experiences a reduction in its diffusion coefficient.^28,29^ In this case, the particle evolves in a static potential landscape *U*(*y*,*z*) and its motion is affected through the coefficients *A*_2_ and *A*_3_ in eq 2. More precisely, one has Δ*T*_eff_ = 0, *D*_*ij*_ = δ_*ij*_D_E_, *A*_1_ = (M – M_w_)­g/(MΓ), *A*_2_ = −[∂*U*/∂*y*]/(MΓ), and *A*_3_ = −[∂*U*/∂*z*]/(MΓ) for such a particle. If one now defines an effective diffusion coefficient D̃, for instance, as $0.5\underset{t\to \infty }{\lim}\left\langle {\left[y_{p}\left(t\right)-y_{p}\left(0\right)\right]}^{2}\right\rangle /t$ in terms of the *y* component of the particle’s position, D̃ would be less than D_E_ = k_B_ *T*_0_/(MΓ). Evidently, the fact that $\mathrm{D̃}<D_{E}$ is *not* a collective (many-particle) effect and, besides, it cannot be interpreted as a reduction in the effective temperature.

The colloidal suspension consists of 200 nm polystyrene spheres with a density of 1050 kg/m^3^ and a mean surface-to-surface distance of 1.8 μm suspended in water in a cm-sized glass cuvette. Unlike experiments involving one single particle or a small number of particles,^1,3,5,26,28–32^ one cannot use imaging and tracking techniques to follow the trajectories of individual particles in a dense, large system of particles. Instead, we use a variant of the coherence-gated dynamic light scattering^33,34^ as depicted in Figure 2.

**Figure 2:**
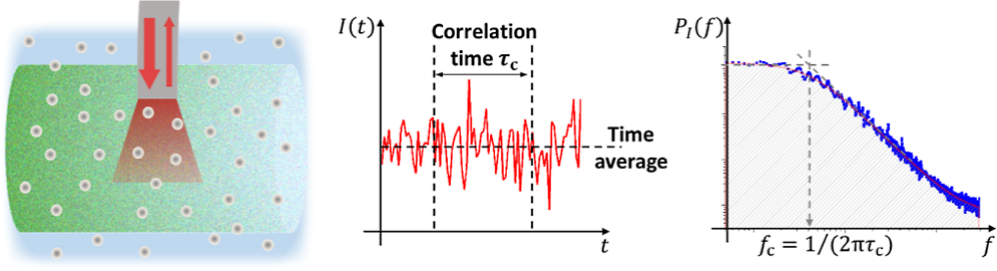
Continuous dynamic characterization of a colloidal system under the combined action of gravity and an optical field (green). A single-mode fiber probe is used to illuminate the sample (thick red arrow) and collect the reflected and scattered light (thin red arrow). Both the local particle number density and the motional temperature are derived from the properties of the detected intensity (see text for details).

For the results presented here, the particles are first uniformly distributed and then kept for a time t_0_ + 0.5 h only under the influence of gravity. After that, the colloidal medium is illuminated from the left by a coherent laser beam of wavelength 532 nm and diameter 4 mm. The effect of t_0_ on the evolution of the particle number density $n\left({\mathrm{r⃗}}_{o},t\right)$ is clearly seen in Figure 3. The reference point ${\mathrm{r⃗}}_{o}$ is the same for both curves; the probe is placed at approximately 1 mm from the left wall of a 1 cm-in-width cuvette, 1 cm from the top air–water interface, and 2 cm from the bottom of the cuvette. Since the probe is positioned in the upper part of the cuvette, the effect of sedimentation would be to decrease the local particle density which is observed in the first 30 min before switching on the green light. In agreement with the discussion of eq 2, the physical system never forgets its history. After turning on the laser at *I*_0_ = 13 W/cm^2^, the evolution of the particle number density depends on *t*_0_. The effect of turning on the laser is evident as an increase in the particle number density. The radiation pressure of the incident beam pushes particles from the left of the fiber tip into the detection volume.

**Figure 3:**
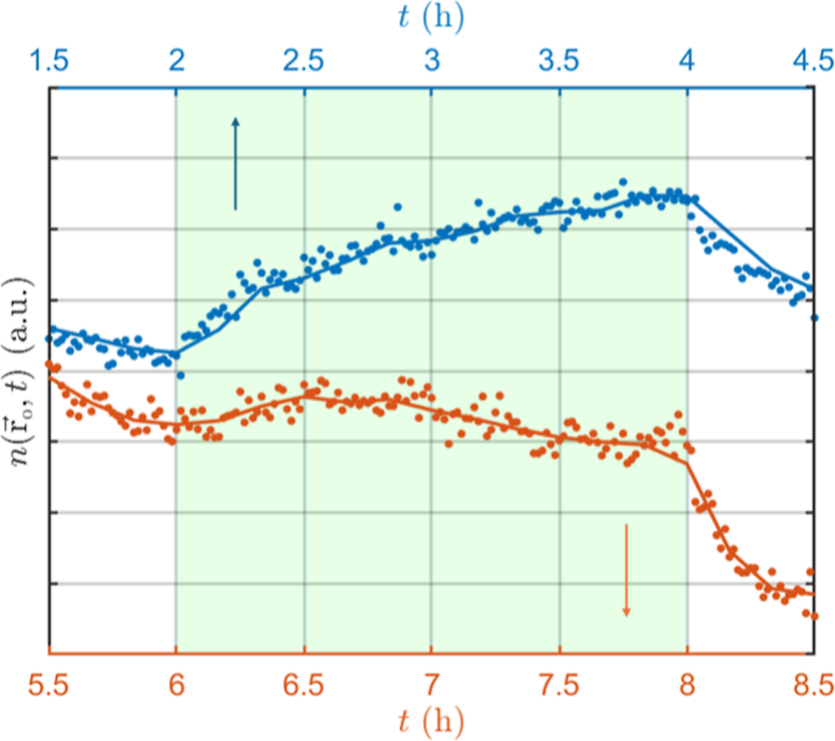
Time evolution of the particle number density $n\left({\mathrm{r⃗}}_{o},t\right)=N\left({\mathrm{r⃗}}_{o},t\right)/V_{o}$ , where *N* denotes the number of particles within the detection volume V_o_. The colloidal system is only under the influence of gravity for 1.5 h (blue) and for 5.5 h (orange). After an additional 0.5 h, the green laser is turned on for a duration of 2 h. As predicted, the evolution of $n\left({\mathrm{r⃗}}_{o},t\right)$ depends on the time elapsed until the optical field is applied.

### Effective Motional Temperature of the Interacting System of Particles

Let us now examine ${\Delta T}_{eff}\left({\mathrm{r⃗}}_{o},t\right)$ . Before the laser is turned on, Δ*T*_eff_ should be zero. However, the blue data points in Figure 4 show fluctuations even before the laser is turned on. The reason is that each data point corresponds to one estimation of the photocurrent PSD based on one time slot of a finite duration Δt < ∞.

**Figure 4:**
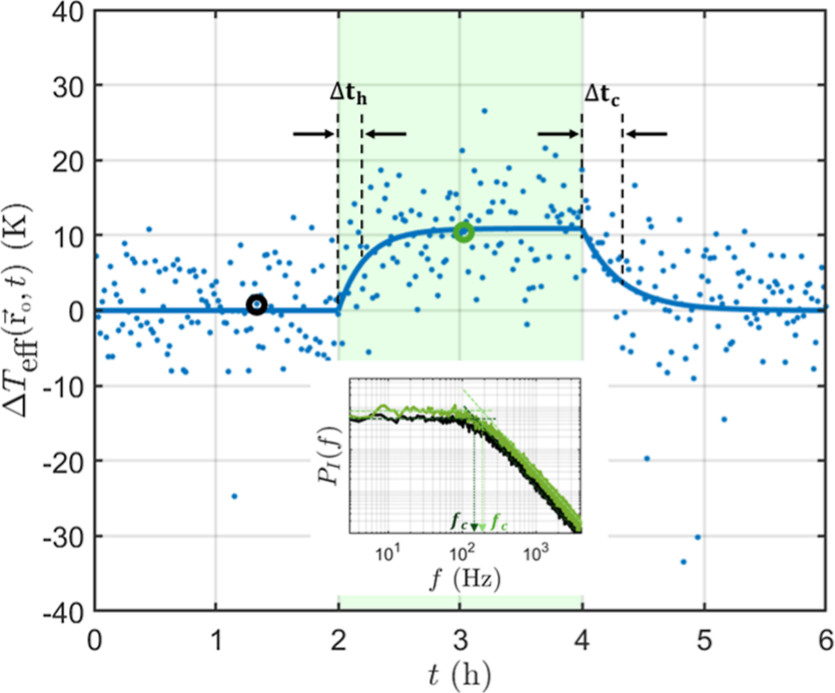
Time evolution of $\Delta T_{eff}\left({\mathrm{r⃗}}_{o},t\right)=T_{eff}\left({\mathrm{r⃗}}_{o},t\right)-T_{0}$ , where *T*_eff_ is the measured motional temperature, and T_0_ is calculated as its time average over 1 h < *t* < 2 h. The green shadow indicates the 2 h during which the green laser is on. The blue line represents the exponential thermal response model fitted to the data points. The inset shows the photocurrent’s PSD for two specific time slots: before (black) and in (green) the presence of the laser field.

After the laser is turned on at I_0_ = 13 W/cm^2^, the experiments reveal a noticeable Δ*T*_eff_ as seen in Figure 4. This is due to the additional random force ${\mathrm{f⃗}}_{sp}$ generated by the time-varying speckle field. As seen in Figure 4, Δ*T*_eff_ gradually increases and reaches a final value of Δ*T*_eff,max_ = 11 K after the laser is turned on. This final value is evaluated as the time average of Δ*T*_eff_ over the last hour in the presence of the laser light (i.e., over 3 h < *t* < 4 h). We note that this large Δ*T*_eff,max_ is not an effect of light absorption, as water’s temperature, which was monitored continuously, showed variations smaller than 2 K over the entire experiment duration.

To better describe the time evolution of Δ*T*_eff_(*t*), the blue line in Figure 4 represents an exponential fit to the data points as follows:

$$\Delta T_{eff}\left(t\right)=\{\begin{array}{ll} 0, & 0\;h<t<2\;h\\ \left(1-e^{-\left(t-t_{1}\right)/{\Delta t}_{h}}\right){\Delta T}_{eff,\max}, & 2\;h<t<4\;h\\ e^{-\left(t-t_{2}\right)/\Delta t_{c}}{\Delta T}_{eff,\max}, & 4\;h<t<6\;h \end{array}$$ 3

where t_1_ and t_2_ denote the times when the laser is turned on and off, respectively. It is noticeable that the heating time Δt_h_ is not equal to the cooling time Δt_c_. For the example shown in Figure 4, one finds that Δt_c_ is approximately one and a half times longer than Δt_h_. In our experiments, we consistently find that the increase of the motional temperature is faster than its decrease. Heating faster than reciprocal cooling has recently been discussed in the context of thermodynamic systems far from equilibrium.^5,7^ Our results indicate that this asymmetry between heating and cooling times also exists in the case of a large-scale, interacting many-body system.

Let us now examine how Δ*T*_eff,max_ depends on the intensity I_0_ of the laser beam. The autocorrelation $R_{sp}\left({\mathrm{r⃗}}_{o},t;\tau \right)$ of each component of the exerted ${\mathrm{f⃗}}_{sp}\left({\mathrm{r⃗}}_{o},t\right)$ on a test particle within the detection volume is $\left[K_{sp}\left({\mathrm{r⃗}}_{o},t\right)/\tau_{sp}\left(t\right)\right]e^{-2\left|\tau \right|/\tau_{sp}\left(t\right)}$ , where both *K*_sp_ and τ_sp_ vary slowly over time when the laser is turned on. The former increases and eventually reaches *K*_sp,max_, which is proportional to Δ*T*_eff,max_, while the latter decreases and reaches τ_sp,min_, which is proportional to 1/(T_0_ + Δ*T*_eff,max_). On the other hand, we expect *R*_sp_(τ) to scale with I_0_^2^, because optical forces scale with I_0_. Therefore, one finds that

4 $$\gamma +\gamma^{2}=\xi I_{0}^{2}$$

where γ denotes Δ*T*_eff,max_/T_0_, and ξ is a constant independent of I_0_. We repeated the experiment for several laser powers and measured γ for each case where Δ*T*_eff,max_ is the average of Δ*T*_eff_ over the last 2 h in the presence of the laser light. The laser remained on for an additional hour in this experiment. The results are summarized in Figure 5, where the continuous line is the fit to eq 4 with ξ being the fitting parameter. The fit error for the plotted curve evaluated as the sum of squared errors (SSE) is in this case 3.26 and less than that of a linear fit γ ∝ I_0_ (SSE = 3.93) or a quadratic fit γ ∝ I_0_^2^ (SSE = 3.62).

**Figure 5:**
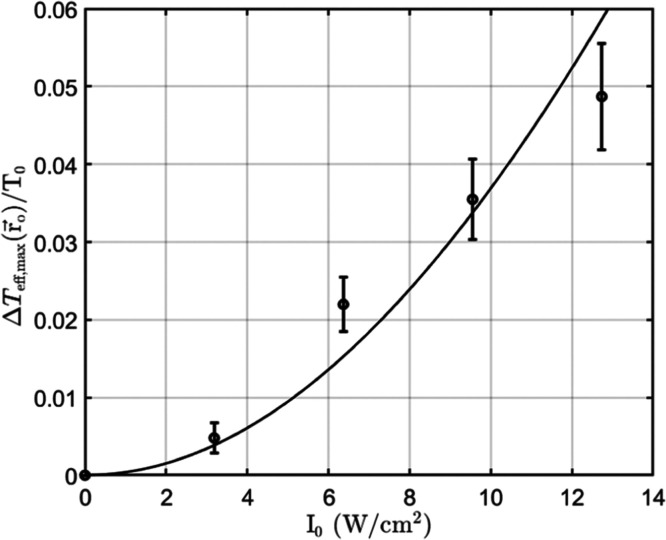
Measured ${\Delta T}_{eff,\max}\left({\mathrm{r⃗}}_{o}\right)/T_{0}$ for different laser intensities. The curve is fitted to the measured data according to eq 3.

## Conclusions

Nonlinear and out-of-equilibrium effects arising in interacting many-body systems are far from being well understood. Because analytical and numerical methods have their own limitations, access to controllable experimental platforms is highly desirable. In this paper, we present one such possible testbed. Our many-body physical system is a dense suspension of nanoparticles under the influence of gravity and an external laser beam.

The particles interact with each other in our system. Each particle experiences an optical force due to the speckle field generated by all other particles. This optical force has an explicit time dependence. The macroscopic physical quantity emerging from the interaction is a motional temperature. Notably, unlike all previous studies,^14,21–28^ the motional temperature introduced here is a collective effect.

Traditional imaging and tracking approaches^1,3,5,27,29–33^ are not suitable for examining dense, three-dimensional colloidal systems such as ours. We used a coherence-gated light scattering procedure to examine particles within a picoliter detection volume.^34,35^ This technique permits detecting both the number density of particles and their motional temperature, which is proportional to their average kinetic energy.

As expected from theory, we observed a gradual and significant increase in the motional temperature after the laser beam was turned on. We observed that the heating time, during which the motional temperature increases after the laser beam is turned on, is considerably smaller than the cooling time, during which the motional temperature decreases back to its initial value after the laser beam is turned off. This observation agrees with the conclusions that have recently been made for one-particle systems.^5,7^ We also theoretically and experimentally derived how the maximum motional temperature changes with laser intensity.

This physical system and experimental setup provide a powerful and unique platform for studying different collective manifestations of interacting, many-body systems. Additional constraints and complementary probing can easily be integrated to study the dynamics of complex matter with multiple degrees of freedom. This could, for instance, unravel generic thermodynamic constraints and examine general quantitative predictions about nonequilibrium systems.^36,37^ It is worth noting that relations such as Jarzynski’s equality^36,38^ and the so-called thermodynamic uncertainty relation^37,39^ hold for nonequilibrium systems with a constant temperature in its traditional thermodynamic sense, while the collective motional temperature of our nonequilibrium many-body system is a type of generalized temperatures and undergoes a time evolution after the external source of energy is turned on.

Lastly, we have shown that colloids can serve as models for a different type of matter that is capable of generating motion and stress. As opposed to passive matter like common solids or liquids, such “active matter” has unique mechanical properties that can be traced back to its constituents’ ability to convert additional energy, stored or imparted from the environment, into collective motion.^40,41^ Our work demonstrates a colloidal model for active matter where the convenient optical tuning allows controlling the macroscopic properties and provides means for exploring the intricate properties of active matter. Colloidal active matter may also open avenues for creating synthetic materials that could mimic properties of living matter, which are typical examples of active matter.^42^ In the present work, the interparticle distances are large enough, i.e., the concentration of colloidal particles is small enough, such that other types of interparticle forces, including electrostatic and hydrodynamic forces,^43^ are negligible. Future work will examine in detail the competition between multiple types of such forces.

## Methods

### Optical Probe

The single-mode fiber (SMF) guides the probe light (λ = 670 nm) toward the sample and also gathers the red photons that are reflected and scattered by the sample and guides them toward a photodetector. The photocurrent provides information about both the particle number density $n\left({\mathrm{r⃗}}_{o},t\right)$ and the motional temperature $T_{eff}\left({\mathrm{r⃗}}_{o},t\right)=T_{0}+\Delta T_{eff}\left({\mathrm{r⃗}}_{o},t\right)$ , where ${\mathrm{r⃗}}_{o}$ denotes the position of the fiber tip. A high spatial resolution is determined by the size of the SMF’s core in the *yz* plane (4 μm) and by the coherence length of the probe light in the *x* direction (approximately 30 μm). In a coherence-gated dynamic light scattering technique, such as the one used in our experiments, the source has a low coherence length. The time resolution of a measurement is limited by the time Δ*t* necessary to evaluate the power spectral density (PSD) of the photocurrent. Typically, we used time slots of duration Δ*t* = 15 s with a sampling time of 5 μs.

$T_{eff}\left({\mathrm{r⃗}}_{o},t\right)$ is determined from the PSD of the photocurrent. In deriving *T*_eff_ for each time slot, we assume that the number *N* of particles within the detection volume does not change significantly during that time slot. This however does not mean that we do not have access to the slight changes that really happen in *N*. In fact, a notable feature of our optical probing technique is its sensitivity to the variations in *N*, which, as explained further, is proportional to the time average of the photocurrent over the time slot.

### Dynamics of the Photocurrent

The electric field of the photons gathered by the SMF can be written as E_ref_ + T_fw_ *E*_s_(*t*), where E_ref_ denotes the field of the photons reflected back due to the water–fiber interface, *E*_s_(*t*) denotes the field of the photons scattered back by the particles in water, and *T*_fw_ denotes the water-to-fiber field transmission coefficient. Note that E_ref_ = (T_wf_ – 1)­E_0_, where E_0_ denotes the field of the photons of the red source before they enter water, and T_wf_ denotes the fiber-to-water field transmission coefficient.

The field *E*_s_(*t*) can be written as $\alpha_{p}E_{inc}\overset{N}{\underset{m=1}{\sum }}e^{i\mathrm{q⃗}\cdot {\mathrm{r⃗}}_{m}\left(t\right)}$ , where E_inc_ denotes the amplitude of the field illuminating the particles in water, ${\mathrm{r⃗}}_{m}\left(t\right)$ denotes the instantaneous position of the m-th particle within the detection volume, and $\mathrm{q⃗}={\mathrm{q⃗}}_{s}-{\mathrm{q⃗}}_{inc}$ is the difference of the wavevector of the scattered light to be gathered by the SMF and the wavevector of the incident red light in water. Given the assumption previously made about the coordinate axes, *E*_s_(*t*) can be written as $\alpha_{p}E_{inc}\sum_{m=1}^{N}e^{-iqx_{m}\left(t\right)}$ , where q is 4πn_w_/λ in terms of λ and the refractive index n_w_ of water. Note that E_inc_ = T_wf_E_0_. Also, the coefficient α_p_, which captures the polarizability of each particle as well as the path attenuation of its scattered light, can be simplified as $\frac{\pi V_{p}}{\mathrm{x̅}\lambda^{2}}\frac{n_{p}^{2}-n_{w}^{2}}{n_{p}^{2}+{2n}_{w}^{2}}$ , where V_p_ and n_p_ denote the particle’s volume and refractive index, respectively, and *x̅* is an average distance between the tip of the SMF and a particle within the detection volume.

The photocurrent *I*(*t*) in response to the photons gathered by the SMF can be written as ${\eta \left|E_{ref}+T_{fw}E_{s}\left(t\right)\right|}^{2}$ , where η captures all photon-to-electrical-energy conversion coefficients, including the photodetector’s efficiency. Therefore, *I*(*t*) reads

5 $$I\left(t\right)=A+BRe\left[\sum_{m=1}^{N}e^{iqx_{m}\left(t\right)}\right]+C\sum_{m=1}^{N}\sum_{m^{\prime }=1}^{N}e^{iq\left[x_{m}\left(t\right)-x_{m^{\prime }}\left(t\right)\right]}$$

where $A=\eta {\left(T_{wf}-1\right)}^{2}{\left|E_{0}\right|}^{2}$ , $B=2\eta \alpha_{p}T_{fw}T_{wf}\left(T_{wf}-1\right){\left|E_{0}\right|}^{2}$ , and $C={\eta \alpha_{p}^{2}T_{fw}^{2}T_{wf}^{2}\left|E_{0}\right|}^{2}$ . Note that B^2^ = 4AC.

### Time Average of the Photocurrent

One can assume that the number *N* of particles within the detection volume changes much slower than the particles’ positions *x*_m_ in eq 5. Therefore, the time average of *I*(*t*′) over a time slot *t* – 0.5Δ*t* < *t*′ < *t* + 0.5Δ*t* can be written as ⟨*I*⟩_Δ*t*_ = A + C*N*(*t*). This means that the time evolution of *N*(*t*) is the same as the time evolution of the measured ⟨*I*⟩_Δ*t*_.

### Power Spectral Density of the Photocurrent

The PSD *P*_*I*_(*f*) of *I*(*t*) is formally defined as the Fourier transform of *R*_*I*_(τ) = ⟨*I*(*t*)*I*(*t* + τ)⟩ – ⟨*I*(*t*)⟩⟨*I*(*t* + τ)⟩ with respect to τ. This definition suggests that *I*(*t*) should be a stationary stochastic process in the sense that the expectation values ⟨*I*(*t*)⟩ and ⟨*I*(*t*)*I*(*t* + τ)⟩ do not change with *t*. Also, assuming that *I*(*t*) is ergodic as well, any expectation value ⟨·⟩ can be considered as a time average $\lim_{\Delta t\to \infty }\left(\int_{t-0.5\Delta t}^{t+0.5\Delta t}\left(\cdot \right)\;dt^{\prime }\right)/\Delta t$ . Therefore, assuming that *I*(*t*) is ergodic, *P*_*I*_(*f*) is equal to ${\left|Ĩ\left(f\right)\right|}^{2}$ , where *Ĩ*(*f*) denotes the Fourier transform of *I*(*t*).

Before the green laser, which generates optomechanical interaction between particles, is turned on, the only nonstationary ingredient of *I*(*t*) in eq 5 is the slowly varying number *N* of particles within the detection volume (see Figure 3). In other words, as far as the dynamics *x*_m_ of each particle in eq 5 is concerned, it is stationary before the green laser is turned on. Each particle has a fixed drift velocity ${\mathrm{x̂}v}_{g}=\mathrm{x̂}\left(M-M_{w}\right)g/\left(M\Gamma \right)$ due to gravity and also does a Brownian motion with a fixed diffusion coefficient D_E_ = k_B_T_0_/(MΓ) at room temperature T_0_ before the green laser is turned on. Therefore, the effective motional temperature *T*_eff_ should be fixed and equal to T_0_ before the green laser is turned on. However, the measured *T*_eff_, which is represented by the blue data points in Figure 4, shows fluctuations over time even before the green laser is turned on. The reason is that each data point in Figure 4 corresponds to one estimation of *Ĩ*(*f*) based on the values of *I*(*t*) in one time slot of a finite duration Δ*t* < ∞.

After the green laser is turned on, the dynamics *x*_m_ of each particle in eq 5 is not stationary anymore because, due to the optomechanical interaction between the particles, each particle does a Brownian motion with a diffusion coefficient *D* = k_B_ *T*_eff_/(MΓ) that changes slowly over time. However, by estimating *Ĩ*(*f*) for individual time slots of the duration Δ*t*, and calculating the moving average of the data points, we can see how *T*_eff_ gradually increases (decreases) after the green laser is turned on (off).

It is worth noting that another effect of the green laser is its radiation pressure that leads to an additional drift velocity ${ẑv}_{rp,m}$ for each particle (i.e., each “m”). As previously explained, *v*_rp,m_ depends on the position of the particle, especially on its *z* coordinate (i.e., *z*_m_).

Let us now see how *T*_eff_ can be deduced from *P*_*I*_(*f*). In view of eq 5, *P*_*I*_(*f*) is equal to the Fourier transform of the sum of two terms with respect to τ,

$$R_{het}\left(\tau \right)=2ACN\left(t\right)\;Re\left\langle e^{iq\left[x\left(t+\tau \right)-x\left(t\right)\right]}\right\rangle$$

and

$$R_{\mathrm{hom}}\left(\tau \right)=C^{2}\left[N^{2}\left(t\right)-N\left(t\right)\right]{\left|\left\langle e^{iq\left[x\left(t+\tau \right)-x\left(t\right)\right]}\right\rangle \right|}^{2}$$

where the subscripts “het” and “hom” refer to “heterodyne” and “homodyne”, respectively. As each particle has a drift velocity v_g_ along the *x* axis and also does a Brownian motion with a diffusion coefficient *D*, the expectation value ⟨*e* ^*I*q[*x*(*t*+τ)–*x*(*t*)]^⟩ in the above equations is equal to $e^{i{qv}_{g}\tau }e^{-q^{2}D\left|\tau \right|}$ .^3^ Therefore, *P*_*I*_(*f*) is the sum of two terms:

$$P_{het}\left(f\right)=2ACN\left(t\right)\frac{f_{c}}{2\pi }\left[\frac{1}{{\left(f-f_{0}\right)}^{2}+f_{c}^{2}}+\frac{1}{{\left(f+f_{0}\right)}^{2}+f_{c}^{2}}\right]$$

and

$$P_{\mathrm{hom}}\left(f\right)=C^{2}\left[N^{2}\left(t\right)-N\left(t\right)\right]\frac{{2f}_{c}}{\pi }\frac{1}{f^{2}+{4f}_{c}^{2}}$$

where *f*_0_ = qv_g_/(2π) and *f*_c_ = q^2^ *D*/(2π). By fitting the measured PSD with the theoretical formula, we can find *f*_c_ and therefore *D*, which is proportional to *T*_eff_. It is worth noting that, as v_g_ is very small (around 1 nm/s), *f*_0_ is around 4 mHz and therefore negligible.

### Horizontal Diffusion vs Vertical Sedimentation

In our system, sedimentation is minimal due to the small size of the particles (R = 100 nm). To quantify this, the relevant time scales of diffusion and sedimentation are compared. The time it takes for a particle to diffuse over its own size t_d_ = R^2^/D_E_ ∼5 ms is much shorter than the time it takes for the particle to settle over its size t_s_ = R/v_g_ ∼90 s. This results in a Peclet number Pe = t_d_/t_s_ ≪1 which characterizes a “Brownian” regime where diffusion dominates over sedimentation.^44^

The velocity of sedimentation for one particle is estimated as follows v_g_ = 2g­(ρ_p_ – ρ_w_)­R^2^/9η ∼9 nm/s, where ρ_p_ = 1050 kg/m^3^ is the density of the particle, and ρ_w_ = 998 kg/m^3^ is the density of the medium. Since the coherence length of the incident light is l_c_ ∼30 μm, the time it takes to empty this vertical distance below the end of the probe is approximately t_empty_ = l_c_/v_g_ ∼7 h. This time is longer than the duration of the measurement. This suggests that the sedimentation process is slow enough that the region directly below the fiber does not become significantly depleted of particles.

Moreover, during the time it takes the particle to sediment vertically, it experiences frequent kicks from the other particles diffusing horizontally, which prolongs the time needed for the region under the probe to empty out. The horizontal spread of the particles due to diffusion can be estimated to be D*_E_t*_empty_ ∼0.06 mm^2^, which is larger than the cross-sectional area of the fiber ∼0.02 mm^2^. This indicates that the horizontal diffusion continuously replenishes the region below the fiber, so any fiber-blocking effect is negligible.

### Error Analysis

To evaluate the uncertainty in the effective temperature measurement, we analyze the dimensionless quantity γ ≡ Δ*T*_eff,max_/T_0_ measured at different laser intensities. Here, Δ*T*_eff,max_ is obtained by averaging Δ*T*_eff_ over the last 2 h of the laser illumination, and T_0_ is the temperature before turning on the light. Each individual measurement γ_*i*_ is extracted from a PSD averaged over a 1 min interval, resulting in Q = 121 data points over the 2 h of illumination. The average of these individual measurements corresponds to a single data point in Figure 5 $\mathrm{\gamma ̅}=\frac{1}{Q}\overset{Q}{\underset{i=1}{\sum }}\gamma_{i}$ . Assuming that all the individual measurements γ_*i*_ have equal uncertainty σ_γ*i*_ = SD­(γ), the uncertainty in γ̅ could be estimated using the Standard Error of the Mean $\sigma_{\mathrm{\gamma ̅}}=\frac{SD\left(\gamma \right)}{\sqrt{Q}}$ where the standard deviation SD­(γ) is $SD\left(\gamma \right)=\sqrt{\frac{1}{Q-1}\sum_{i=1}^{Q}{\left(\gamma_{i}-\mathrm{\gamma ̅}\right)}^{2}}$ .

However, in our case, individual measurements γ_*i*_ may not have the same uncertainty. Each Δ*T*_eff_ value has its own uncertainty, which is directly related to the uncertainty in determining the corner frequency *f*_c_. This is in addition to the errors encountered in the averaging process; therefore, a more detailed error analysis is required.

First, there is the error in determining the corner frequency *f*_c_ from a typical PSD measurement. The shape of the PSD is assumed to be a Lorentzian; however, noises from different sources affect the measurement in both the low- and high-frequency ranges. At low frequencies, the PSD is influenced by the finite duration of the measurement. Longer measurement time is needed to get a flat horizontal line in the low-frequency region. At high frequencies, the PSD is more susceptible to noise from external sources. To minimize these influences, we impose asymptotic Lorentzian behavior at both ends of the spectrum. In the low-frequency limit *f* → 0, the PSD is a constant, while in the high-frequency limit *f* → ∞, it decays with a slope of −2 in the log–log representation. Instead of applying a full Lorentzian fit, which requires fitting with two parameters at the same time, we extract the corner frequency by finding the intersection of two linear segments: a horizontal line in the low-frequency region and a line with a slope of −2 in the high-frequency region. Each of these segments has only one fitting parameter which improves precision and avoids the complications of a multiparameter nonlinear fit.

In the low-frequency range (1–50 Hz), the PSD is fitted with a horizontal line corresponding to the average *a* over the low frequency range up to 50 Hz. In the high-frequency range (800–8000 Hz), the PSD is fitted with a line logP­(*f*) = −2log*f* + *b*, where *b* is the fitting parameter. The intersection of the lines defines the corner frequency $f_{c}=\sqrt{\frac{10^{b}}{a}}$ . Each of the parameters *a* and *b* has its own uncertainty, σ_*a*_ and σ_*b*_, respectively. If the errors in *a* and *b* are uncorrelated, the uncertainty in the corner frequency *f*_c_(*a*,*b*) can be estimated using the first-order error propagation formula $\sigma_{f_{c}}=\sqrt{{\left(\frac{\partial f_{c}}{\partial a}\right)}^{2}\sigma_{a}^{2}+{\left(\frac{\partial f_{c}}{\partial b}\right)}^{2}\sigma_{b}^{2}}=\sqrt{{\left(\frac{1}{2a}f_{c}\right)}^{2}\sigma_{a}^{2}+{\left(\frac{\ln10}{2}f_{c}\right)}^{2}\sigma_{b}^{2}}$ .

The uncertainty in *a* is the standard error of the mean, $\sigma_{a}=\frac{SD\left(a\right)}{\sqrt{\varphi }}=\frac{1}{\sqrt{\varphi }}\sqrt{\frac{1}{\varphi -1}\sum_{k=1}^{\varphi }{\left(P\left(f_{k}\right)-a\right)}^{2}}$ , where φ = 50, corresponding to the number of data points in the frequency interval (1–50 Hz).

The uncertainty in the intercept *b* of a least-squares fit to a straight line relates to the overall fit uncertainty $\sigma_{fit}=\sqrt{\frac{1}{\sqrt{\varphi }}\underset{f_{800}<f_{j}<f_{8000}}{\sum }{\left(\log P\left(f_{j}\right)+2\log f_{j}-b\right)}^{2}}$ through a coefficient that depends on the spread of the independent variable of the fit function, log*f*,^4^ $\sigma_{b}=\frac{\sigma_{fit}}{\sqrt{\underset{f_{800}<f_{j}<f_{8000}}{\sum }{\left(\log f_{j}-\overset{®}{\log f}\right)}^{2}}}$ , where *f*_*j*_ are the discrete frequency bins, *l* is the number of bins, and $\overset{®}{\log f}$ is the mean of log*f_j_* in the high frequency range (800 Hz - 8000 Hz).

The uncertainty in γ_i_ relates to the error in estimating the corner frequency for the *i*-th PSD curve as $\sigma_{\gamma_{i}}=\frac{1}{f_{c_{0}}}\sigma_{f_{c_{i}}}$ where $f_{c_{0}}$ is the corner frequency corresponding to T_0_, evaluated as the average corner frequency during the hour prior to turning on the green laser, and $\sigma_{f_{c_{i}}}$ is the uncertainty of the corner frequency estimated from the error propagation formula. Then, the error in γ̅ is determined using the weighted standard error of the mean $\sigma_{\mathrm{\gamma ̅}}=\sqrt{\frac{1}{\overset{Q}{\underset{i=1}{\sum }}\left(1/\sigma_{\gamma_{i}}^{2}\right)}}$ .

To account for the possible increase in water temperature, assumed to scale with the laser intensity, we add a systematic uncertainty to $\sigma_{\mathrm{\gamma ̅}}$ . Since the upper bound of the water temperature increase was measured to be ∼2 K at the highest laser intensity I_0.max_, the total uncertainty becomes $\sigma_{tot}=\sqrt{\sigma_{\mathrm{\gamma ̅}}^{2}+{\left(\left(\frac{2}{T_{0}}\right)\left(\frac{I_{0}}{I_{0,\max}}\right)\right)}^{2}}$ . This total uncertainty encapsulates the statistical uncertainty from the measurement in addition to the systematic uncertainty because of the water heating. This procedure provides a more reliable estimate of the error associated with each data point in Figure 5.
